# Herpes Simplex Virus-2 Esophagitis in a Young Immunocompetent Adult

**DOI:** 10.1155/2016/7603484

**Published:** 2016-04-19

**Authors:** Deepak K. Kadayakkara, Angela Candelaria, Ye Eun Kwak, Caroline Loeser

**Affiliations:** ^1^Department of Medicine, Bridgeport Hospital-Yale New Haven Health, Bridgeport, CT 06610, USA; ^2^Department of Gastroenterology, Bridgeport Hospital-Yale New Haven Health, Bridgeport, CT 06610, USA

## Abstract

Herpes simplex esophagitis (HSE) is commonly identified in immunosuppressed patients. It is rare among immunocompetent patients and almost all of the reported cases are due to HSV-1 infection. HSV-2 esophagitis is extremely rare. We report the case of a young immunocompetent male who presented with dysphagia, odynophagia, and epigastric pain. Endoscopy showed multitudes of white nummular lesions in the distal esophagus initially suspected to be candida esophagitis. However, classic histopathological findings of multinucleated giant cells with eosinophilic intranuclear inclusions and positive HSV-2 IgM confirmed the diagnosis of HSV-2 esophagitis. The patient rapidly responded to acyclovir treatment. Although HSV-2 is predominantly associated with genital herpes, it can cause infections in other parts of the body previously attributed to only HSV-1 infection.

## 1. Introduction

Herpes simplex virus (HSV) has been recognized with increased frequency as an opportunistic invader of the esophagus in immunosuppressed or severely ill patients as a result of primary infection or more commonly viral reactivation [1]. Patients at greater risk include those with human immunodeficiency virus (HIV) infection, underlying malignancy, burns, organ transplantation, and immunosuppressive therapy or systemic corticosteroids [2]. In immunocompetent patients, HSE is typically primary infection and is self-limited. HSE is rare, with only a few cases reported and limited published reviews [3]. Canalejo et al. reviewed patients with HSV esophagitis diagnosed with histopathology or tissue viral culture of which HSV-1 was identified in 27 cases and HSV-2 was identified in 1 case. A clinician needs to have a high index of suspicion in a young healthy adult who presents with an acute onset of dysphagia, odynophagia, and epigastric pain [4]. Patients may or may not present with prodromal symptoms such as malaise, fever, pharyngitis, and cough [4, 5].

## 2. Case Presentation

**Figure 1:**
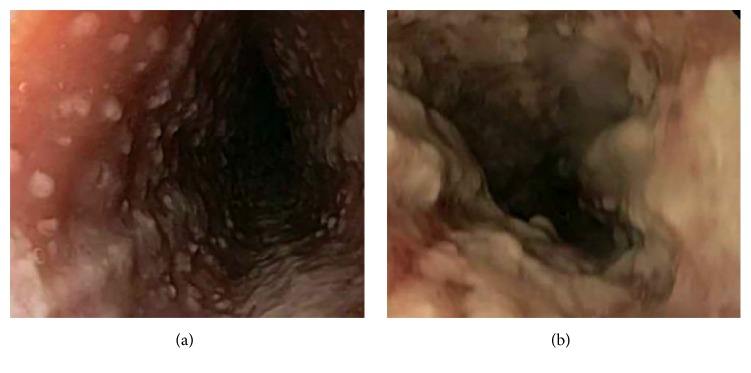
Endoscopy shows multitudes of small ulcers with normal intervening mucosa (a) and areas of confluent plaque-like lesions (b) in the distal esophagus.

**Figure 2:**
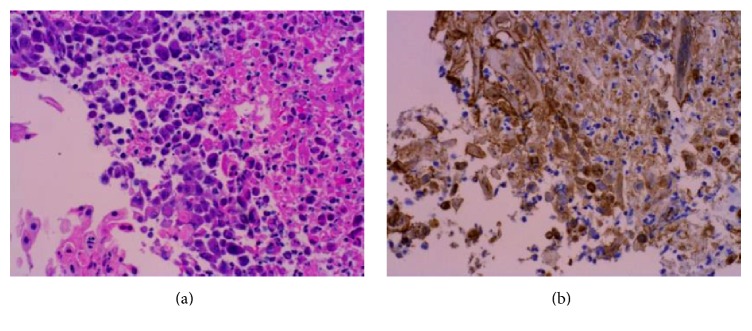
(a) H&E of the esophagus showing mucosal necrosis with smudgy nuclear chromatin and multinucleation, highly characteristic of HSV infection (400x). (b) Showing positive immunohistochemistry for HSV (400x).

We present a case of a 28-year-old gentleman who presented to the hospital with four days of progressive dysphagia, odynophagia, and epigastric pain and two days of subjective fever. He was unable to tolerate oral intake and had lost approximately 8 pounds over the last several days. He had been in a sexual relationship with one partner for six months and was unaware of any potential exposure to HSV. His past medical history is significant for asthma controlled without steroids. At admission he was febrile with a temperature of 102.8 and tachycardia of 110/min. There were no oral or pharyngeal lesions. He had tenderness in the epigastric area, but no palpable masses or hepatosplenomegaly. Laboratory workup was significant only for atypical lymphocytes noted on smear. Esophagogastroduodenoscopy (EGD) revealed small ulcers with overlying exudate with normal intervening mucosa with some areas of confluence in the lower and middle third of the esophageal mucosa (Figure 1). The confluent areas were concerning for severe candida esophagitis. He was empirically started on IV fluconazole. Brushings and biopsies were positive for multinucleated giant cells with eosinophilic intranuclear inclusions. Immunostains of the biopsy samples were positive for HSV (Figure 2). Immunostains for fungus and CMV were negative. Laboratory testing showed a significantly high level of HSV type 2 IgM titer. His HIV status was negative and evaluation for an immunosuppressive state was unremarkable. His initial treatment of IV fluconazole was changed to IV acyclovir in response to the biopsy results and he responded quickly to the treatment. He was discharged with oral acyclovir for a fourteen-day treatment.

## 3. Discussion

HSE is common in immunosuppressed patients. HSE is rare, but distinct entity in immunocompetent patients. [2, 3]. The most common presentation includes acute onset of odynophagia, dysphagia, retrosternal chest pain (e.g., GERD symptoms), and fever. Less frequent symptoms include a prodromal syndrome, weight loss, and upper respiratory symptoms such as cough [2, 6]. In one review of 56 patients approximately 20% developed herpetic orolabial lesions [2]. Other complications were upper gastrointestinal bleeding (5.6%), of which one case was esophageal perforation [2, 3]. It is important to have a high index of suspicion of HSE in any healthy patient (including the pediatric and elderly population) who present with an acute onset of odynophagia, fever, and retrosternal pain without a clear etiology [1–3, 6]. The classic presentation of HSE on endoscopy exam shows evidence of disease in the distal (68.3%) or midesophagus [3]. Lesions are often multiple, discrete, or coalescent small ulcers. The ulcers may be superficial, appearing punched-out or volcano-like in appearance [2, 3].

HSV-1 is most commonly associated with HSE, although HSV-2 infections were reported rarely [2]. HSV-2 infections were predominantly associated with genital herpes; however, it has shown to cause rarely meningitis, pulmonary infections, and esophagitis. Most of the infections are seen in immunocompromised individuals. The case presented in this report is another example of rare but possible infection of HSV-2 in esophagus of a healthy adult. What predisposes these patients to HSV-2 infection is not entirely clear. In immunocompetent patients, treatment with oral acyclovir for a duration of 7–10 days is recommended instead of the traditional 10–14-day treatment recommended for immunocompromised patients. In our patient, due to the extensive nature of the disease, we treated him for 14 days.

This case also presented with a unique challenge for endoscopic diagnosis of HSE. Initially, due to the white-plaque-like raised appearance of the lesions, candida esophagitis was suspected. There were more characteristic discrete nummular lesions hidden around the large plaque-like lesions. HSV-2 was ultimately diagnosed by immunohistochemistry for HSV and IgM antibodies for HSV-2. IgM/IgG antibodies were not detected for HSV-1. Although tissue immunohistochemistry and PCR for HSV-2 would have been alternate methods to confirm the diagnosis, due to the technical difficulties, we were not able to perform them. However, we believe that the evidence is compelling in this case to confirm the diagnosis of HSV-2 esophagitis.
